# Differences in Educational Effects of Basic Life Support Course in a Manufacturing Company by the Number of Previous Participations

**DOI:** 10.7759/cureus.81541

**Published:** 2025-03-31

**Authors:** Yuchi Maeda, Masaaki Matsunaga, Yupeng He, Nanpei Hattori, Koji Ishikawa, Atsuhiko Ota

**Affiliations:** 1 Department of Public Health, Fujita Health University School of Medicine, Toyoake, JPN; 2 Department of Human Resource Management, Nagoya Health Management Group, Mitsubishi Heavy Industries, Ltd., Nagoya, JPN

**Keywords:** automated external defibrillator, cardiopulmonary resuscitation, confidence, multiple training, workplace

## Abstract

Objectives

The objective is to clarify whether conducting a basic life support (BLS) course in the workplace enhances employees’ confidence in and knowledge of BLS, and to assess if the impact diminishes for those who participate repeatedly.

Methods

We studied 299 manufacturing company employees who attended a 60-to-90-minute BLS course. Participants were divided into three groups based on the number of previous attendance sessions: none, one or two, and three or more. A self-administered questionnaire was used to assess their levels of confidence in checking responses, chest compressions, and the use of an automated external defibrillator (score range: 0-5) and knowledge (0-7) before and after the BLS course. We examined the change in scores using a two-way repeated analysis of variance.

Results

Confidence and knowledge scores increased for all participants after the BLS course (p < 0.001). Time-by-group interactions among the three groups for confidence and knowledge scores were all significant. The lower the number of previous courses attended, the greater the increase in scores. The increase in confidence and knowledge scores was significantly greater in those who had never taken a BLS course than in those who had taken it three or more times.

Conclusions

Our findings suggest that the BLS course was highly effective for participants who had never taken a BLS course. It may be necessary to formulate course content specifically tailored to those who have repeatedly attended the course, as opposed to merely perpetuating the same course content.

## Introduction

In Japan, workforce aging is expected to increase the incidence of cardiovascular diseases and the demand for emergency medical services [1]. The proportion of individuals aged 65 years and above in the total labor force has surpassed 10%, exhibiting a persistent upward trend over an extended period [2]. In cardiopulmonary arrest, studies have demonstrated that the survival rate declines by 6% every minute when defibrillation is delayed [3]. Consequently, the immediate initiation of cardiopulmonary resuscitation (CPR) and electrocardioversion with an automated external defibrillator (AED) is considered the most crucial factor for enhancing the clinical outcomes of cardiac arrest injuries [4,5].

In the context of occupational safety, companies are legally obligated to ensure the well-being of their workforce. Moreover, employers in Japan are formally responsible for ensuring the safety of workers. However, in practice, this obligation is often delegated to managers [6]. As the use of AEDs by the general public has become more prevalent, the concept of duty of care considerations, including AEDs, has changed [7,8]. In cases where individuals do not take action to save lives, legal proceedings have occurred, and the duty of safety considerations may be interpreted more broadly in the future. This could result in litigation arising from instances of cardiac arrests in the workplace. Notably, there is a growing need for employees to acquire basic life support (BLS) skills and take appropriate action if colleagues experience cardiac arrest [9]. Occupational health workers are defined as individuals involved in the healthcare of employees in a company and assist in fulfilling their duty of care for safety [10]. Therefore, occupational health workers need to assume the role of instructors and facilitate BLS courses, thereby ensuring the development and maintenance of a company's first-aid system.

As BLS courses in the workplace are conducted during working hours, participants must be both efficient and highly educated. In a previous study, a lack of confidence and insufficient knowledge were reported as reasons for failure to perform CPR [11,12]. Conversely, higher levels of confidence and knowledge acquisition were associated with a greater probability of performing BLS without hesitation [13].

In a study of non-medical staff working at a university hospital, a survey of students and faculty reported that confidence in the technique increased after the course as the number of courses taken increased [14,15]. However, few studies have examined the educational effects of BLS courses on manufacturing industry employees. Here, a questionnaire survey was administered to manufacturing employees who had attended a BLS course before and after the course. It was hypothesized that the educational effects, that is, the level of confidence and improvement in knowledge of the techniques, would differ depending on the number of times they had attended BLS courses.

## Materials and methods

Participants

The participants in this longitudinal study were employees at a particular workplace. They were selected from those who voluntarily participated in BLS courses conducted by occupational health workers between July 2023 and February 2024. Individuals who answered and submitted a questionnaire survey after completing the course were selected. The exclusion criteria included those who did not respond to any of the items and those who participated in multiple courses of FY2023. In total, 301 individuals participated in the course and completed the questionnaire, resulting in a response rate of 100%. After the exclusion of two individuals who attended the course multiple times in FY2023, the final analysis included 299 participants.

Outline of BLS course

In the context of the safety initiatives undertaken by the Aircraft Manufacturing Department of a particular plant in FY 2023, the safety manager requested that the health office conduct BLS courses. Occupational health workers serving as instructors had previously attended a three-hour general lifesaving course and received instructions on CPR for adults and the use of AEDs. Approximately 20 individuals participated in each course. The duration of the course was between 60 and 90 minutes, with equal time allocated for classroom instruction and practical skills training. The classroom curriculum included a diagram illustrating the AED layout in factories, a flowchart outlining emergencies, points to note when using an AED for women, case studies of past incidents, and a sample of the sequence of BLS procedures. Throughout the year, the same educational materials based on the Japanese resuscitation guidelines were used to standardize the teaching methods. Practical training included hands-on experience with chest compressions and AED operations using two resuscitation training dolls. All participants had the opportunity to perform chest compressions, and some were engaged in AED operations. At the end of the course, the participants were invited to respond to a questionnaire.

Survey method

Questionnaires were administered before and after the BLS course. The questionnaires were answered anonymously, and the surveys aimed to determine participants’ levels of confidence in BLS techniques and their knowledge of BLS before and after the course. The participants were asked to complete a pre-course questionnaire before the BLS course began and a post-course questionnaire at the end of the course. Questionnaires were collected on-site immediately after the course.

Study variables

The survey items included fundamental attributes such as job title, age, sex, the number of times participants attended a BLS course, the number of years since their last BLS course, the presence of family members in the medical field, and their experience in a collapsed situation. The survey also assessed confidence levels in various BLS techniques, including checking responses, chest compressions, and AED use. Finally, participants were asked to indicate their knowledge of BLS.

The level of confidence in each technique was assessed using the following three questions: “Can you confirm whether the patient is responsive (conscious)?,” “Can you perform chest compressions?,” and “Can you use an AED?” These question items were based on previous studies [14,16] to ensure content and criterion validity. For each question item, participants were instructed to respond on a 6-point scale ranging from 0 (no) to 5 (yes). The scale was structured as follows: 0 = 0, 1 = 1, 2 = 2, 3 = 3, 4 = 4, and 5 = 5. Higher scores indicated higher BLS confidence. The knowledge acquisition included seven questions. The questionnaire is attached to the Appendices. Questions were presented in a three-way multiple-choice format. Correct answers were assigned a value of 1 point, and the total score was evaluated on a 7-point scale. A higher score indicated a higher level of BLS knowledge. In consultation with the coauthors, we developed questions about the level of reasonable knowledge based on previous research [15,17,18]. The percentage of participants who achieved the upper limit of the measurement range was calculated to assess the presence or absence of a ceiling effect.

Statistical analyses

The participants were classified into three groups based on the number of times they attended BLS courses: those who had never attended BLS courses (none-course group), those who had participated in one or two courses (one or two course group), and those who had attended three or more courses (three or more course group).

A one-way analysis of variance was performed to compare the mean confidence and knowledge scores for each of the before-course and after-course sessions between the groups. Tukey’s post-hoc test was used for multiple comparisons, with the significance level set at p < 0.05. Changes in confidence and knowledge scores were evaluated using a two-way repeated analysis of variance to determine whether they differed over time (before and after the course) and among the three groups. Multiple comparisons were performed for the time-by-group interactions between any two of the three groups. The F- and P-values were adjusted using the Greenhouse-Geisser correction for degrees of freedom (df) when the sphericity assumption was violated. Notably, Holm's method was used to compare pre-to-post score differences between groups. Moreover, the McNemar test was applied to assess the change in the percentage of correct answers to the BLS knowledge questions before and after the course, with the significance level set at p < 0.05. Calculations were performed using IBM SPSS Statistics version 29.0 (IBM Corp., Armonk, NY).

## Results

The participants’ fundamental attributes are listed in Table 1. They were classified according to the number of times they had previously attended BLS courses. The classification was as follows: 94 were in the none-course group, 145 in the one-or-two-course group, and 44 in the three-or-more-course group. Of these, 96.0% (287/299) were male, 4.0% (12/299) were female, 32.8% (98/299) were in their 30s, and 32.8% (98/299) were in their 40s.

**Table 1:** Main characteristics of the subjects (n=299). BLS: basic life support.

Variables		n (%)
Job title		
	Manager	121 (40.5)
	Other than manager	174 (58.2)
	Missing	4 (1.3)
Age group		
	18-29	33 (11.0)
	30-39	98 (32.8)
	40-49	98 (32.8)
	50-59	54 (18.1)
	60 years or above	16 (5.4)
Gender		
	Male	287 (96.0)
	Female	12 (4.0)
	Other	0 (0.0)
Progress since BLS training		
	None-course	94 (31.4)
	One-or-two-course	145 (48.5)
	Three-or-more-course	44 (14.7)
	Missing	16 (5.4)
Number of years since last participation (excluding 0 participation)		
	Less than 5 years	41 (13.7)
	5 years or more and less than 10 years	56 (18.7)
	More than 10 years	68 (22.7)
	Missing	40 (13.3)
Presence of medical personnel in the family		
	Yes	69 (23.1)
	No	229 (76.6)
	Missing	1 (0.3)
Experience of encountering a situation where a person has fallen		
	Yes	67 (22.4)
	No	230 (76.9)
	Missing	2 (0.7)

Table 2 presents confidence and knowledge scores by group and before the course. Before the course, there was a significant difference in the means between the groups in checking responses (F (2, 279) = 5.555, p = 0.004), and the three-or-more-course group showed a higher level (mean: 3.48) of checking responses than did the none-course group (p = 0.005). For chest compressions, there was a significant difference in the mean values between the groups (F (2, 279) = 23.811, p < 0.001), with significant differences among all groups. Furthermore, there was a significant difference in the means between the groups when using an AED (F (2, 279) = 43.184, p < 0.001) and between all groups (p < 0.001 for each). In terms of knowledge scores, there was a significant difference in the means between the groups (F (2, 280) = 4.903, p = 0.009), and the three-or-more-course group showed a higher level (mean: 3.55) than did the none-course group (p = 0.006). After the course, there were no significant differences in the mean values between the groups. The mean (standard deviation) post-training confidence scores for all participants were 4.73 (0.59) for checking responses, 4.67 (0.61) for chest compressions, 4.72 (0.56) for AED use, and the mean knowledge score was 6.56 (0.73) for mean knowledge score. The proportion of participants who attained the maximum score for checking responses, chest compressions, AED use, and knowledge exceeded 60% in all cases, suggesting a ceiling effect.

**Table 2:** Confidence and knowledge scores by group and before the course. AED: automated external defibrillator. *P-values were calculated with a one-way analysis of variance, which was used to assess the variances in confidence and knowledge scores among the three groups before the course. **P-values were calculated with Tukey’s post-hoc test.

	Group	Mean (standard deviation)	F-value	P-value*	Multiple comparisons: P-value**	
Checking responses (on a scale of 0 to 5)	None-course	2.57 (1.590)	5.555	0.004	0 time < 1-2 times	0.051
	One-or-two-course	3.06 (1.631)			1-2 times < 3 or more times	0.275
	Three-or-more-course	3.48 (1.267)			0 times < 3 or more times	0.005
Chest compression (on a scale of 0 to 5)	None-course	0.89 (1.213)	23.811	< 0.001	0 time < 1-2 times	< 0.001
	One-or-two-course	1.69 (1.361)			1-2 times < 3 or more times	0.001
	Three-or-more-course	2.48 (1.303)			0 times < 3 or more times	< 0.001
AED use (on a scale of 0 to 5)	None-course	0.78 (1.313)	43.184	< 0.001	0 time < 1-2 times	< 0.001
	One-or-two-course	1.71 (1.368)			1-2 times < 3 or more times	< 0.001
	Three-or-more-course	3.02 (1.285)			0 times < 3 or more times	< 0.001
Knowledge scores (on a scale of 0 to 7)	None-course	2.70 (1.516)	4.844	0.009	0 time < 1-2 times	0.319
	One-or-two-course	2.99 (1.429)			1-2 times < 3 or more times	0.075
	Three-or-more-course	3.55 (1.591)			0 times < 3 or more times	0.006

**Figure 1:**
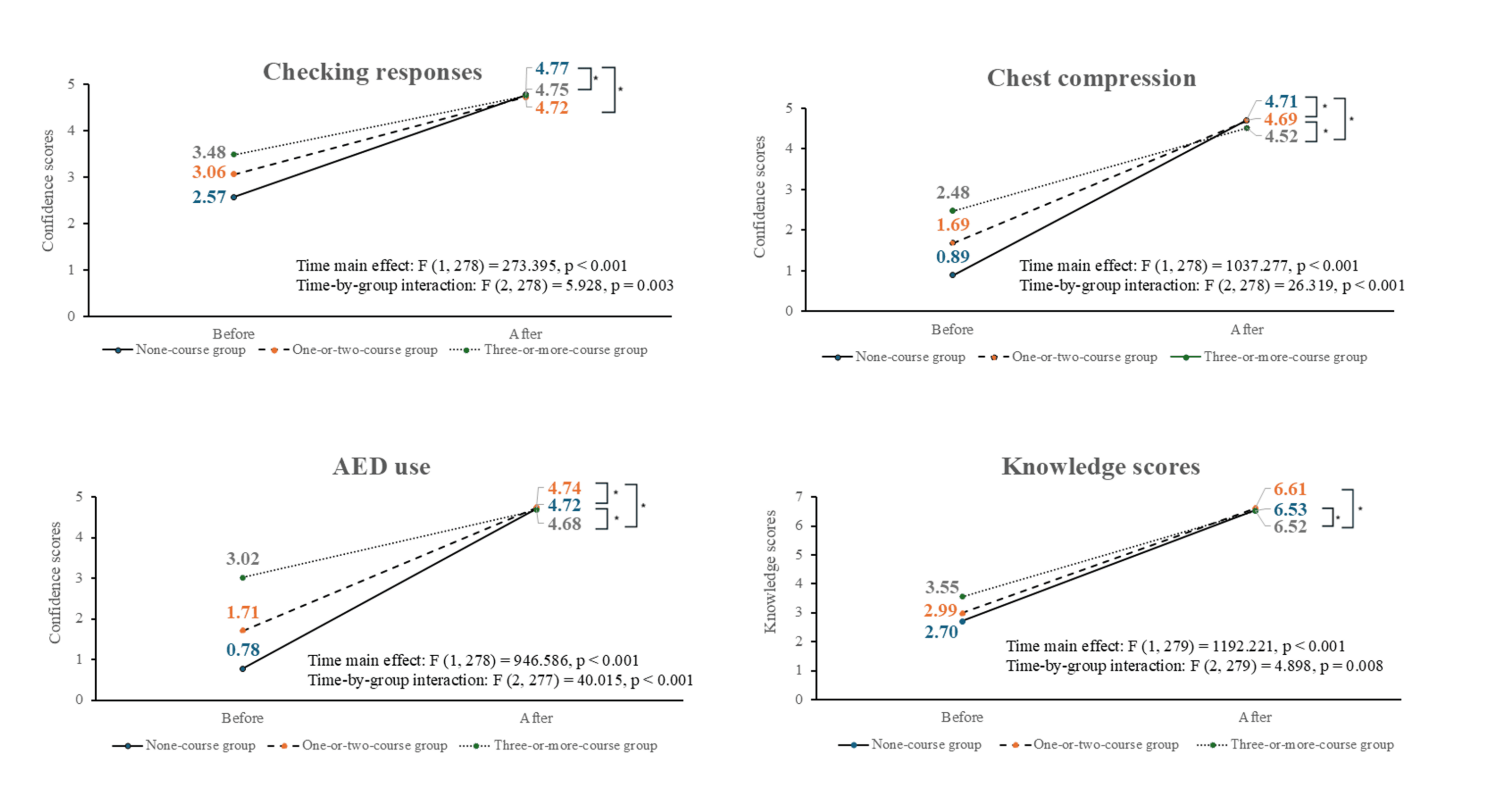
Confidence and knowledge scores by group before and after the course. AED: automated external defibrillator. Time main effects and time-by-group interaction effects are displayed above each graph. Asterisks (*) indicate significant pre-to-post score differences between groups based on Holm-adjusted significance thresholds (α = 0.05).

Confidence and knowledge scores by group and pre- and post-course are shown in Figure 1. For the confidence scores, there was a significant time-by-group interaction for checking responses (F (2,278) = 5.928, p = 0.003). Multiple comparisons showed that the change from pre-score to post-score in the none-course group significantly differed from that in the one-or-two-course group (F (1, 235) = 5.755, p = 0.017) and the three-or-more-course group (F (1, 136) = 11.812, p < 0.001). For chest compressions, there was a significant time-by-group interaction (F (2, 278) = 26.319, p < 0.001). Multiple comparisons demonstrated that the pre-to-post change significantly differed between the none-course group and the one-or-two-course group (F (1, 235) = 19.703, p < 0.001), between the none-course group and the three-or-more-course group (F (1, 136) = 56.283, p < 0.001), and between the one-or-two-course group and the three-or-more-course group (F (1, 185) = 15.672, p < 0.001). There was a significant time-by-group interaction for AED use (F (2, 277) = 40.015, p < 0.001). Multiple comparisons showed that the change from pre-score to post-score in the none-course group significantly differed from that in the one-or-two-course group (F (1, 234) = 21.836, p < 0.001) and the three-or-more-course group (F (1, 135) = 82.426, p < 0.001). The pre-to-post change was also significantly different between the one-or-two-course group and the three-or-more-course group (F (1, 185) = 33.604, p < 0.001). There was a significant time-by-group interaction for knowledge scores (F (2, 279) = 4.898, p = 0.008). Multiple comparisons showed that the change from pre-score to post-score in the three-or-more-course group significantly differed from that in the none-course group (F (1, 136) = 7.873, p = 0.006) and one-or-two-course group (F (1, 186) = 7.534, p = 0.007).

Table 3 presents the number of correct answers and the percentage of correct answers to questions on BLS knowledge before and after the course. Regardless of the number of prior participations, the percentage of correct answers for all questions was significantly higher after the training than before. Even in the three-or-more-course group, the percentage of correct answers was less than 50% before the course for questions regarding how to call for confirmation of consciousness, tempo of chest compressions, and cardiac arrest necessitating AED shock. After training, the percentage of correct answers was less than 90% for questions regarding the appropriate course of action in the case of a collapsed person and cardiac arrest necessitating shock with an AED.

**Table 3:** Number of correct answers and percentage of correct answers to the questionnaire on the basic life support knowledge (before and after the course). AED: automated external defibrillator. *P-values were calculated with McNemar test.

No.	Question	Percentage of correct answers								
		None-course group (n = 94)			One-or-two-course group (n = 145)			Three-or-more-course group (n = 44)		
		Before (%)	After (%)	P-value*	Before (%)	After (%)	P-value*	Before (%)	After (%)	P-value*
1	There is a person who has collapsed. What would you do?	54.3	85.1	< 0.001	68.1	89.6	< 0.001	63.6	86.4	0.002
2	When checking the consciousness of a collapsed person, how do you call it out loud while performing the following actions?	23.4	100	< 0.001	28.5	97.9	< 0.001	34.1	100	< 0.001
3	Within how many seconds do you check for breathing?	66.3	94.6	< 0.001	60.8	95.1	< 0.001	73.8	92.9	0.021
4	Which chest compression rate should you use?	20.4	91.4	< 0.001	19.4	95.8	< 0.001	37.2	93.0	< 0.001
5	How deep should chest compressions be in an adult victim?	43.0	100	< 0.001	44.4	100	< 0.001	53.5	100	< 0.001
6	Which cardiac arrest requires an AED shock?	17.2	88.2	< 0.001	23.6	86.1	< 0.001	29.5	88.6	< 0.001
7	The hiccups seen in injured patients immediately after cardiac arrest. What should you do if you have a breathing problem?	49.5	94.5	< 0.001	52.1	96.5	< 0.001	67.4	97.7	< 0.001

## Discussion

The following hypothesis was tested in this study: the number of BLS training courses attended would affect the level of confidence and knowledge of the techniques of checking responses, chest compressions, and using an AED before and after the course. As hypothesized, the change in confidence and knowledge scores before and after the intervention in the none-course group was significantly higher than that in the three-or-more-course group. This finding suggests that the confidence and knowledge scores of the none-course group increased significantly after the course, while the confidence and knowledge scores of the three-or-more-course group did not increase as much.

Even in the three-or-more-course group, techniques and knowledge associated with relatively low retention rates were identified. The percentage of correct answers after the course was less than 90% for the question of what actions should be taken if an individual falls. These results are consistent with those of a previous study [17], suggesting that acquiring this knowledge is relatively challenging. In the future, it will be essential to emphasize that bystander safety must be prioritized. Furthermore, the performance of the participants on questions about cardiac arrest necessitating shock with an AED was less than 90% after the course. The percentage of correct answers before the course was less than 30%, indicating that this was the most challenging question. In a study involving medical students, approximately 90% correctly identified VF as a rhythm requiring shock [18], revealing a dissociation in knowledge between medical students and adult laypersons. Even in the three-or-more-course group, the percentage of confidence scores for chest compressions was less than 50% before the course, which was lower than those for checking responses and AED use. Given the rapid decline in CPR skills over the past few months [19-21], it is important to encourage employees to participate in repeat courses and provide regular information about BLS to ensure updating of their knowledge.

Here, confidence levels showed a marked increase after the course for each technique regardless of the number of previous experiences, which is inconsistent with the results of previous studies [14,15]. In previous studies, the duration of the course was 30-45 minutes, whereas that of this course was 60-90 minutes. A systematic review found that students' CPR skills improved significantly as they spent more time practicing with a resuscitation training doll [22], indicating that this may apply to working adults. Fear of infection is a factor in the reluctance to perform BLS [23,24]. In fact, following the Japanese resuscitation guidelines, which suggest that chest compressions are the primary focus of CPR training [25], we omitted instructions on artificial respiration in practical training. Instead, the emphasis was placed on chest compressions and AED use. In the academic department, instructors addressed the need to enhance awareness among participants by incorporating AED placement diagrams in factories, flowcharts for emergencies, and case studies of past incidents. Furthermore, a significant proportion of the participants were male, and they were instructed on the precautions to take when using an AED in women. Importantly, reluctance to perform CPR was attributed to concerns about legal repercussions [23,26]. The instructor further noted that, in principle, the use of an AED on a female patient for lifesaving purposes is legally protected. Feedback was shown to increase participants' confidence [21,27]. Instructors provide real-time feedback on the rhythm and depth of chest compressions, as well as posture during chest compressions. The bystander effect has been demonstrated to be reduced in situations where bystanders are acquainted with each other [28-30], thereby facilitating the performance of BCPR in the workplace. We believe that this phenomenon provides a high return on investment. Specifically, it is speculated that participants are more likely to attend the course with a positive attitude, such as “I want to protect my fellow workers,” which may have led to the high educational effect of this course. Considering the knowledge scores, the proportion of accurate responses increased to approximately 90%, regardless of the number of prior experiences. Notably, a statistically significant difference in knowledge scores was observed before and after the training course, which is consistent with the findings of previous studies [31-33]. These results suggest that training courses enhance knowledge acquisition.

The results of the self-administered questionnaire indicate that confidence in each technique improved after the training course. Although the questionnaire survey was conducted anonymously, it is possible that social desirability bias was present in the participants' answers to the confidence questions. Consequently, the enhancement in confidence levels after the course could have been overestimated. We did not objectively evaluate whether the chest compression technique improved. A more objective method of measurement should be introduced in future studies. A previous study [16] reported an association between self-confidence and chest compression technique, and we believe that content and criterion validity are ensured for the questions related to self-confidence. On the other hand, the questions related to knowledge were developed in consultation with the co-authors concerning previous studies [15,17,18], so content validity may be ensured to some extent. However, the other validity and reliability of the assessment of the subjects’ knowledge of BLS were not adequately confirmed. Consequently, only measurement errors could be observed. Additionally, a ceiling effect was observed for both confidence and knowledge scores after training, with responses exhibiting a bias toward the upper end of the scale. Consequently, post-training confidence and knowledge scores may not accurately reflect true changes in participants' performance. The reasons for the ceiling effect included the ease of the knowledge questions, the fact that participants were asked to respond immediately after the course, and the fact that the knowledge questions were easy to answer because of the three-way selection process.

The subjects of this study were workers engaged in a certain manufacturing industry and were sufficiently representative of the entire transportation machinery and equipment manufacturing industry, with more than 1,000 employees. This study included 287 men and 12 women, with a mean age of 42. Notably, the sample was male-dominated, which may limit the validity of the results for discussing the educational effects of gender differences. According to Japan's Employment Status Survey for 2022, among employees working in the transportation machinery and equipment manufacturing industry with 1,000 or more employees, approximately 630,000 are male, and 90,000 are female, with a gender ratio of 9:1. In terms of age, the 35-39 age group exhibited the largest number of employees, at approximately 930,000. The participants voluntarily enrolled in the study. Our findings indicate that increased participation leads to heightened levels of confidence and knowledge acquisition before the course. This suggests that the study group was not unique in its capacity to learn, regardless of the number of courses attended. The importance of repeated BLS training courses was indicated, which is consistent with existing findings [14 ,15]. Specifically, the three-or-more-course group exhibited a higher level of confidence in the use of AED before the course compared to the none-course group and the one-or two-course group, indicating that the participants may have been aware of their ability to use AED if they followed the instructions. However, irrespective of the number of times the participants attended the course, their levels of confidence and knowledge acquisition were lower before the course than after. Future research should focus on maintaining participants’ confidence and knowledge after attending the course.

The present study has several limitations. Primarily, given that two occupational health workers instructed the course, variations in teaching methods may have introduced errors in the educational outcomes. However, given that the educational materials were standardized, it is unlikely that this had a significant impact. Furthermore, participants were divided into groups based on the number of previous BLS courses they had attended; nonetheless, the specific types of courses and the amount of time spent on these courses were not clearly defined. In addition, although the immediate educational effects of training were observed, long-term effects are not yet known and require further study. Finally, the subjects of the present study were selected from a single large company with more than 1,000 employees. To confirm the external validity of the present results, our findings need to be duplicated in different types of workers and occupational settings, including small and medium-sized enterprises. For example, it was reported that individuals with higher levels of education were more likely to perform CPR in an emergency [12,24] and were more knowledgeable about the BLS [26].

The findings of this study indicate the need to adjust the content of BLS courses according to participants' prior experience with BLS courses. To enhance the efficacy of education for employees who have participated in BLS courses three or more times, courses can be provided with suitable content based on prior BLS skill acquisition. Another example is the use of peer education by employees who have participated in BLS courses three or more times. For instance, these employees could serve as instructors following lectures on instructional methods provided by occupational health workers. This is attributable to the limited number of occupational health workers who serve as instructors in BLS courses. Previous research has shown that preparation and instruction facilitate learning [34]. Moreover, studies have demonstrated that there is no difference in teaching effectiveness between peers and professional instructors [35,36].

## Conclusions

We examined the relationship between the number of previous BLS courses taken and educational effects by conducting questionnaire surveys before and after the course. We found a significant time-group interaction between participants who had never taken a BLS course and who had taken a BLS course three or more times in terms of changes in their confidence and knowledge of BLS before and after the course. In other words, participants with no prior experience of BLS courses demonstrated the most significant enhancement in confidence and knowledge of BLS before and after the course, indicating the efficacy of the training. However, more research is needed to determine long-term educational effects. As the number of participants with prior experience of BLS courses increased, their confidence in each technique (particularly AED use) and knowledge of BLS were higher before the course, suggesting the importance of repeated BLS course attendance. However, the changes in confidence and knowledge acquisition before and after the course were relatively limited for those who had attended BLS courses three or more times, suggesting that it may be necessary to develop course content specifically for repeat attendees rather than simply repeating the same course content.

## Appendices

**Table 4:** Sample questionnaire (pretest and post-test) (English translation). AED: automated external defibrillator, CPR: cardiopulmonary resuscitation. Credits: Yuchi Maeda.

No.	Question	Options
1	There is a person who has collapsed. What would you do?	1. Rush to the scene immediately 2. Check the safety of your surroundings and rush to the scene 3. Call for help
2	When checking the consciousness of a collapsed person, how do you call it out loud while performing the following actions?	1. Tapping both shoulders 2. Tapping one shoulder 3. Tapping on the head
3	Within how many seconds do you check for breathing?	1. 3 seconds 2. 10 seconds 3. 15 seconds
4	Which chest compression rate should you use?	1. 60–80 compressions per minute 2. 80–100 compressions per minute 3. 100–120 compressions per minute
5	How deep should chest compressions be in an adult victim?	1. Approximately 1 cm 2. Approximately 3 cm 3. Approximately 5 cm
6	Which cardiac arrest requires an AED shock?	1. The heart is quivering in small increments 2. The heart is beating weakly 3. The heart is not beating at all
7	The hiccups seen in injured patients immediately after cardiac arrest. What should you do if you have a breathing problem?	1. Interrupt chest compressions 2. Continue observation 3. Perform CPR immediately
